# Diagnosis, Evaluation and Treatment of Pulmonary Arterial Hypertension in Children

**DOI:** 10.3390/children5040044

**Published:** 2018-03-23

**Authors:** Benjamin S. Frank, D. Dunbar Ivy

**Affiliations:** Section of Cardiology, Department of Pediatrics, University of Colorado School of Medicine and Children’s Hospital Colorado, Aurora, CO 80045, USA; dunbar.ivy@childrenscolorado.org

**Keywords:** pulmonary arterial hypertension, pulmonary hypertension, pediatrics, review

## Abstract

Pulmonary Hypertension (PH), the syndrome of elevated pressure in the pulmonary arteries, is associated with significant morbidity and mortality for affected children. PH is associated with a wide variety of potential underlying causes, including cardiac, pulmonary, hematologic and rheumatologic abnormalities. Regardless of the cause, for many patients the natural history of PH involves progressive elevation in pulmonary arterial resistance and pressure, right ventricular dysfunction, and eventually heart failure. In recent years, a number of pulmonary arterial hypertension (PAH)-targeted therapies have become available to reduce pulmonary artery pressure and improve outcome. A growing body of evidence in both the adult and pediatric literature demonstrates enhanced quality of life, functional status, and survival among treated patients. This review provides a description of select etiologies of PH seen in pediatrics and an update on the most recent data pertaining to evaluation and management of children with PH/PAH. The available evidence for specific classes of PAH-targeted therapies in pediatrics is additionally discussed.

## 1. Introduction

**Figure 1:**
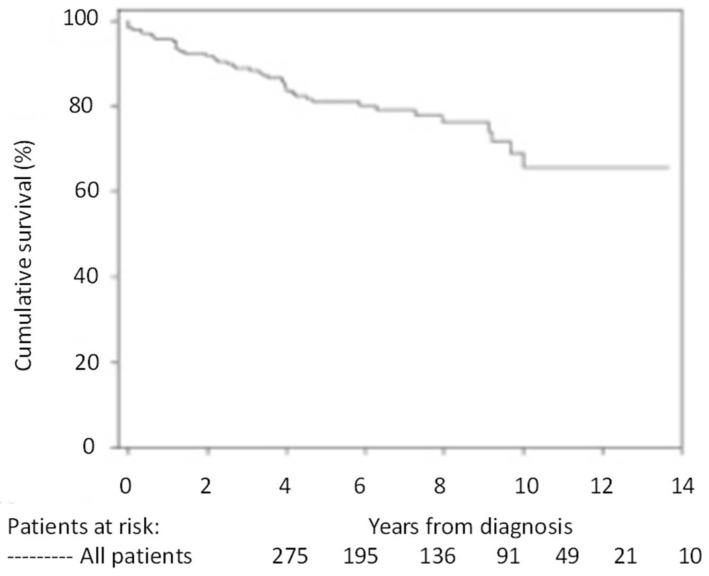
Kaplan–Meier curves showing the survival pediatric pulmonary arterial hypertension (PAH) patients compiled at 3 pulmonary hypertension (PH) centers (Denver, New York, Netherlands): 1-, 3-, 5- and 7-year transplantation-free survival rates were 96%, 89%, 81% and 79%, respectively [4].

Untreated, pulmonary arterial hypertension (PAH) in children carries a poor prognosis. In the 1991 NIH (National Institute of Health) registry study, the median untreated survival for children after diagnosis of idiopathic PAH (IPAH) was 10 months compared to 2.8 years for adults [1]. In 1999, a study by Barst et al. showed that survival for children with severe IPAH who were candidates for intravenous prostacyclin but were unable to be treated with this therapy was poor (45% and 29% at 1 and 4 years, respectively) [2]. Recent advances in understanding the pathobiology of IPAH and new treatment options have resulted in marked improvement in the prognosis for children with PAH (Figure 1) [3,4].

While children and adults with PAH are similar in some respects, many key differences are seen [5]. Across ages, if left untreated, elevation of pulmonary arterial pressure and resistance will lead to right ventricular failure, clinical deterioration and death. Unlike what is seen among adult patients, however, pediatric pulmonary hypertension is intrinsically linked to lung growth and development [6]. Because of this, the young child with pulmonary hypertension has greater potential for pulmonary vascular disease reversal, particularly in the population of patients with PH secondary to lung disease.

Although pediatric-specific evidence is lacking, medical management of children based on clinical experience follows a similar algorithm to that of adults treated with IPAH [3,7,8]. For patients whose disease progresses despite maximal medical therapy, the surgical options include lung transplantation or a Potts shunt—creation of a left pulmonary artery to descending aorta connection in an effort to facilitate right-to-left shunting [9]. This procedure can provide effective palliation in carefully selected children with end-stage pulmonary vascular disease and suprasystemic PAH [10].

## 2. Definition

**Figure 2:**
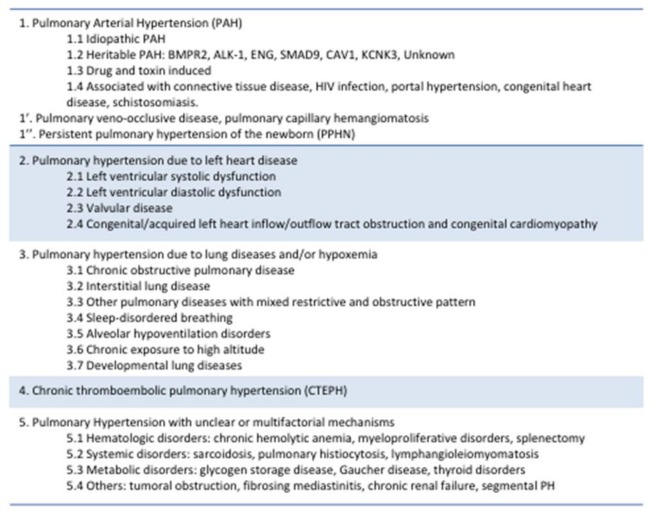
Updated classification of pulmonary hypertension based on recommendations from the 5th World Symposium on Pulmonary Hypertension in Nice, France, 2013. Adapted from [12].

Similar to adults, pulmonary hypertension (PH) in children is defined as a mean pulmonary arterial pressure (PAP) greater than 25 mmHg at rest. The subset of PH patients classified as having PAH additionally demonstrate a pulmonary capillary wedge pressure less than 15 mmHg and an increased pulmonary vascular resistance (greater than 3 Woods units × M^2^) [3,11]. To cohort PH patients into groups by pathophysiology, the Nice classification can be reasonably applied to both adults and children (Figure 2) [3,12]. In younger children, for whom a normal systemic blood pressure is far lower than for older children or adults, the ratio of pulmonary artery mean pressure to systemic artery mean pressure can further describe the severity of PAH, with a significant elevation being greater than 0.5.

An exception to the above definition is the single-ventricle congenital heart disease population with Glenn/Fontan physiology. Because patients who are status post-Fontan palliation rely on passive pulmonary blood flow, pulmonary hypertensive vascular disease complicates the course for many patients despite mean PAP less than 25 mmHg. In such patients, even very mild elevation in pulmonary artery pressure and vascular resistance (PVR) can lead to circulatory failure [6].

## 3. Epidemiology

**Figure 3:**
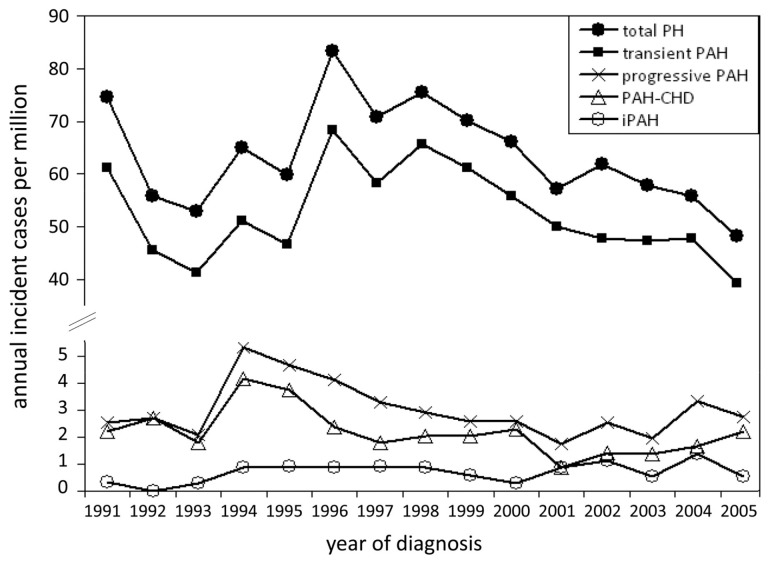
Annual incidence rates for pediatric pulmonary hypertension. PH indicates pulmonary hypertension; PAH, pulmonary arterial hypertension; PAH-CHD, PAH associated with congenital heart defects; and IPAH, idiopathic PAH [14].

National registries from the United Kingdom, the Netherlands and Spain have all shown a lower incidence for IPAH in children compared to adults. The incidence of IPAH in the national registry from the United Kingdom was 0.48 cases per million children per year and the prevalence was 2.1 cases per million [13]. In the Netherlands, annual incidence and point prevalence averaged 0.7 and 2.2 cases per million children, respectively (Figure 3) [14]. Likewise, in the Spanish registry the incidence and prevalence were 0.49 and 2.9 cases per million children, respectively [15]. Recent national database studies from the United States have additionally suggested an increasing prevalence of hospitalized children with PAH as a co-morbidity [16,17].

## 4. Prognosis

**Figure 4:**
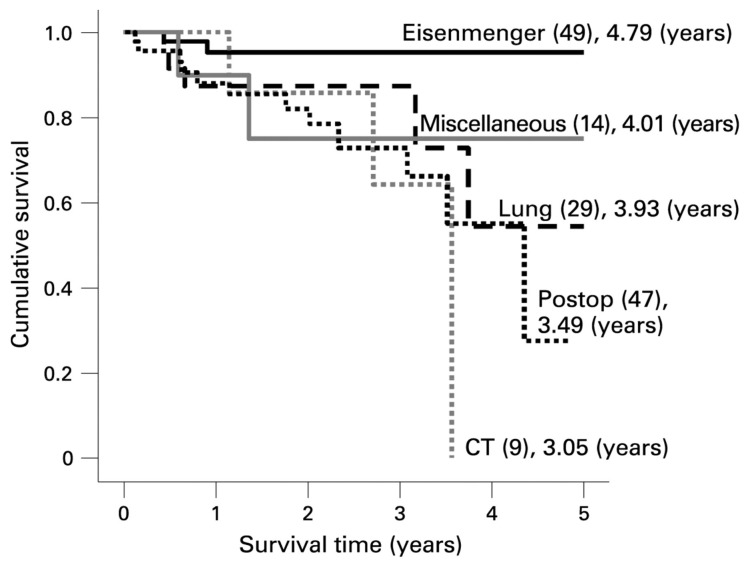
Survival curves for the subgroups within the associated pulmonary arterial hypertension (APAH) group from the UK pulmonary hypertension service. The number in each group (brackets) and the predicted survival out of a possible 5 years is depicted. Note the worse survival for children with post-operative congenital heart disease [18].

As the population of children with PAH is highly heterogeneous, prognosis varies widely. PAH associated with congenital heart disease (CHD) comprises a particularly heterogeneous population. For many children with CHD and a systemic-to-pulmonary shunt, transient PAH is seen pre-operatively with complete resolution after early shunt correction. This group has an excellent prognosis. However, a small subset of patients with CHD develop progressive PAH early in their course that persists after surgical repair. This set of patients has a particularly poor prognosis, worse than those with PAH and unrepaired CHD (Figure 4) [18]. It is not currently possible to precisely predict which patients will resolve their PAH and which patients will progress after anatomic repair. However, it is known that progressive CHD-PAH occurs more frequently in children with genetic syndromes; children with genetic abnormalities comprised 30% of patients with CHD-PAH in a recent analysis [19,20].

## 5. Highlighted Causes of Pulmonary Hypertension (PH)

### 5.1. Heritable Pulmonary Arterial Hypertension (PAH)

Abnormalities in bone morphogenetic protein receptor type 2 (*BMPR2*) are the most commonly identified mutations in children and adults with PAH [21,22,23,24,25,26]. The pattern of inheritance for those with heritable PAH and a *BMPR2* mutation is autosomal dominant with variable penetrance of the PAH phenotype by gender—14% for males and 42% for females [27]. Among adult patients with heritable PAH, *BMPR2* mutations can be identified in ~75% [21]. In pediatric PAH groups, however, results of genetic testing are more variable. Grunig et al. found no *BMPR2* mutations in 13 children with IPAH [23]. However, Harrison et al. found that 22% of children with IPAH or CHD-PAH had a disease-causing mutation [24]. Recently implicated as causative of PAH, *ALK-1* and *TBX4* mutations are common as well [28]. While the gene mutations for both *ALK-1* and *TBX4* can be inherited in an autosomal dominant fashion, the gene penetrance and potential epigenetic modifying factors are not yet well described. In a Japanese study, children with IPAH were approximately as likely to have a *BMPR2* mutation (17%) as an *ALK-1* mutation (12%) [29]. Most recently, Levy et al. found no mutations in any of 23 patients with CHD-PAH but disease-causing mutations in 30% of patients with IPAH (12% *BMPR2*, 10% *ALK-1*, 8% *TBX4*) [28]. Advanced gene-sequencing methods have facilitated the discovery of additional, less common gene mutations implicated among those with PAH (*SMAD-9*, *CAV1*, *KCNK3*, *EIF2AK4*) [21,30,31,32,33].

### 5.2. Single-Ventricle Circulation

In the patient with single-ventricle physiology who has completed Fontan palliation, flow to the pulmonary circulation occurs via direct vena cava to pulmonary artery connections without an intervening pumping chamber. This circulation strategy relies on several factors for adequate cardiac output: anatomically unobstructed pulmonary arterial blood flow and venous drainage, low pulmonary artery pressure (PAP) and PVR, low ventricular end diastolic pressure, and adequate systolic single-ventricular function.

Pulmonary vascular resistance plays a key role in the outcome of the single-ventricle patient [34]. Altered pulmonary perfusion leads to poor pulmonary blood flow and, therefore, low cardiac output. Retrospective studies have demonstrated that mean PAP > 15 mmHg, transpulmonary gradient > 8 mmHg, and indexed pulmonary vascular resistance (PVRI) > 2.5 Wood U × m^2^ are risk factors for Fontan failure, protein-losing enteropathy, and plastic bronchitis [35,36,37]. For Fontan patients who develop these clinical complications, pulmonary vascular disease due to muscular thickening and asymmetric intimal fibrosis of the pulmonary arteries has been implicated [38]. Supporting the theory that Fontan patients can have pulmonary arterial disease despite mean PAP below 25 mmHg, treatment with the PDE-5 inhibitor sildenafil has, in small series, been shown to improve oxygen saturation, exercise capacity, protein-losing enteropathy, and plastic bronchitis [39,40,41]. Additionally, blood concentration of the vasoconstrictor peptide endothelin-1 is increased in Fontan patients [42]. Several studies have suggested improvement in exercise tolerance with endothelin receptor antagonists [43,44,45].

### 5.3. Bronchopulmonary Dysplasia

Bronchopulmonary dysplasia (BPD) is the chronic lung disease associated with prematurity. Advances in neonatal care have improved survival of extremely premature infants but morbidity from BPD is significant, and PH is diagnosed in up to 20% of preterm babies [46]. Although significant PH has been associated with mild BPD, increasing PH severity typically shows a positive correlation with increasing BPD severity. Echocardiographic signs of elevated pulmonary artery pressure are often seen as early as seven days of life and, when present, are associated with higher risk of late PH [47]. In this population, PH is thought to result from a combination of increased vascular tone, hypertensive remodeling, ventilator-induced lung injury, and an underdeveloped pulmonary artery vascular bed. Clinical risk factors for PH in this population include lower gestational age, small-for-gestational age birth weight, oligohydramnios, preeclampsia, prolonged duration of mechanical ventilation, and prolonged oxygen therapy [46,47,48,49]. While PH can resolve or improve as some premature infants grow, persistence and severity of PH are associated with significant mortality: one recent study of premature neonates showed only 53% survival two years after diagnosis of severe, persistent PH [50]. Mesenchymal stromal cells are thought to be lung protective in this group, and pre-clinical animal studies of targeted cell therapy for this population have yielded promising results [51]. Recent guidelines have been published to help standardize provider approach to the diagnosis, monitoring, and management of PH in this particular population [52].

## 6. Standard Evaluation

Recent guidelines on the diagnosis and management of children with PH have helped standardize evaluation and treatment of affected children [53]. A complete evaluation for all possible causes of PH is required before the diagnosis of IPAH is made. Certain diseases that are common in the adult population but less likely to be discovered in children (such as connective tissue diseases and chronic thromboembolic pulmonary hypertension) should still be excluded. Lung biopsy is rarely performed but may be helpful to confirm or exclude entities such as pulmonary veno-occlusive disease (PVOD), pulmonary capillary hemangiomatosis, pulmonary capillaritis, hypersensitivity pneumonitis, and alveolar capillary dysplasia (ACD) when clinical suspicion indicates. Recent genetic insights may allow for less-invasive diagnosis of PVOD (*EIF2AK4*) or ACD (*FOXF1*), further mitigating the need for lung biopsy in those patients. Cardiac catheterization is required to rule out subtle congenital heart disease and to determine right atrial pressure, pulmonary arterial pressure, pulmonary vascular resistance, and reactivity to acute vasodilator testing [54].

Echocardiography is a very useful, non-invasive screening tool to evaluate patients for whom a clinical suspicion of PH exists [55]. The echocardiogram can document cardiac anatomy, right ventricular size and function, left ventricular systolic and diastolic function, morphology and function of valves, the presence or absence of pericardial effusion, and the presence or absence of any shunt lesions. Doppler interrogation of tricuspid valve insufficiency velocity can be used noninvasively to estimate the right ventricular systolic pressure. Pulmonary valve insufficiency is also frequently seen, and characteristics of the pulmonic regurgitant flow velocity can be used to estimate the pulmonary artery diastolic and mean pressures [56].

**Figure 5:**
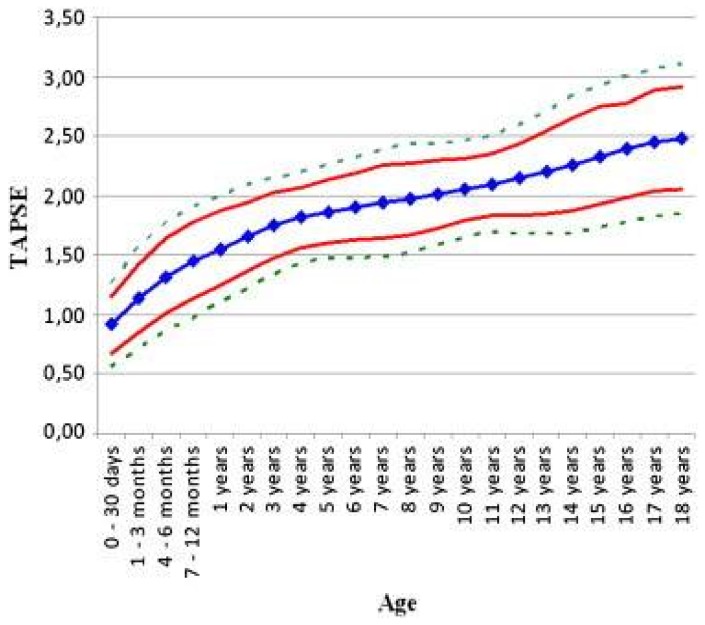
Normal values for tricuspid annular plane systolic excursion (TAPSE) by age [61].

Although challenging due to the geometry of the right ventricle (RV), a qualitative assessment of RV function is also important. Several measures are available to attempt to quantify the degree of RV dysfunction including the Tei index, (myocardial performance index), RV ejection fraction, RV fractional area change and the tricuspid annular plane systolic excursion (TAPSE) [53,57,58,59,60,61,62,63]. Normal values for TAPSE in children have recently been published and should serve as a reference for children with PAH (Figure 5) [61]. A recent study additionally suggested that 3-dimensional echocardiography may improve assessment of RV function compared to 2-dimensional echocardiography alone [64].

**Figure 6:**
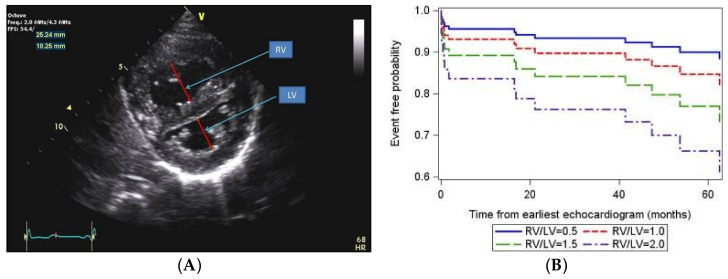
(**A**) Parasternal short axis view of the right and left ventricles (RV/LV) at the level of the papillary muscles. The RV/LV ratio is derived from RV diameter and LV diameter at end-systole. RV/LV end-systolic ratio is predictive of outcome; (**B**) Estimated survival curves for four possible RV/LV ratios estimated from the Cox varying coefficients regression corresponding to a hazard ratio of 2.49 for RV/LV ratio [66].

**Figure 7:**
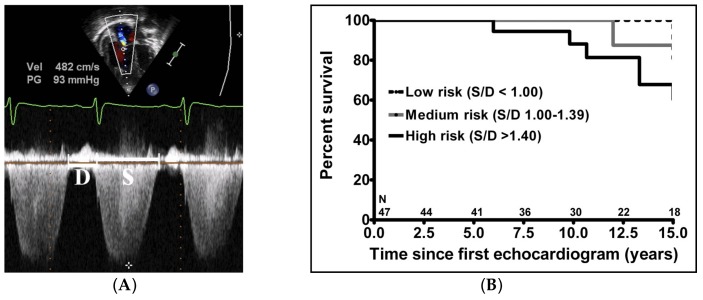
The systolic (S) to diastolic (D) time ratio from tricuspid regurgitation velocity can be measured as an indicator of right ventricular function. (**A**) Measurement of the S/D ratio from a continuous wave Doppler spectrogram; (**B**) An increase in the S/D ratio predicts worse outcome in children with PAH. [68].

**Figure 8:**
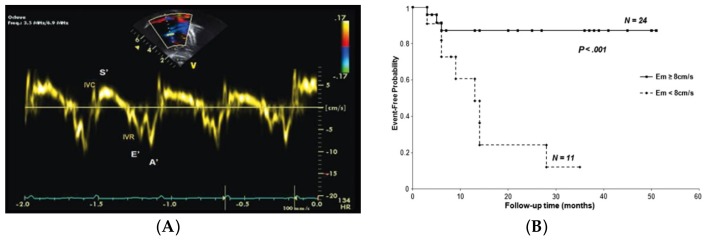
(**A**) A tissue Doppler spectrogram of the right ventricle at the lateral annulus of the tricuspid valve demonstrates the myocardial systolic wave (S’, reflecting the systolic longitudinal movement of the RV) and two diastolic waves (early diastolic (E’) and late diastolic (A’), which reflect the diastolic function of the ventricle); (**B**) E’ velocity less than 8 cm/s is predictive of poor outcome in pediatric IPAH [72].

Recent data have additionally identified echocardiographic markers that can be used to track disease severity and, in some cases, predict clinical outcomes [65]. The ratio of right ventricle to left ventricle (LV) size at end systole is a strong predictor of outcome (Figure 6) [66]. An increasing RV/LV systolic ratio is associated with an increasing hazard for a clinical event (hazard ratio, 2.49; 95% confidence interval, 1.92–3.24). The presence of a pericardial effusion is rare in children, but when present, suggests a poor prognosis [62,67]. As PAH progresses and RV function worsens, the systolic portion of the cardiac cycle lengthens leading to an increase in the systolic to diastolic ratio. The S/D ratio is higher in PAH patients than in controls (1.38 +/− 0.61 vs. 0.72 +/− 0.16, *p* < 0.001), and is associated with worse echocardiographic RV fractional area change, worse catheterization hemodynamics, shorter 6-min walk distance, and worse clinical outcomes independent of pulmonary resistance or pressures (Figure 7) [68,69,70]. Tissue Doppler imaging (TDI) directly measures myocardial velocities and has been shown to be a good measure of RV and LV systolic and diastolic function. In recent pediatric studies, right ventricular TDI velocity was lower in children with PAH compared to healthy controls [71,72] Moreover, tricuspid diastolic velocity (E’) had a significant inverse correlation with right ventricular end-diastolic pressure and mean pulmonary arterial pressure. Cumulative event-free survival rate was significantly lower when tricuspid E’ velocity was ≤8 cm/s (log-rank test, *p* < 0.001, Figure 8) [72]. As the right ventricle contracts primarily in a longitudinal fashion, RV longitudinal strain is a powerful tool to predict clinical outcome in adults with PAH [73]. Its role in evaluating pediatric patients with PAH remains incompletely understood. Finally, function assessment by 3-dimensional echocardiography correlates well with cardiac MRI in children with congenital heart disease [74] and is being evaluated in children with pulmonary hypertension.

Several ancillary tests are additionally useful to evaluate functional status and trend disease severity in PAH patients. The 6-min walk (6MW) test has been used clinically for many years and is a feasible test to quantify sub-maximal exercise in developmentally able children over 7 years of age. Normal values for 6MW distance (6MWD) for children have recently been published [75,76,77,78]. In general, children with PAH tend to walk further than their adult counterparts with the same WHO functional class. Large registry studies have not shown 6MWD to be predictor of survival [13,79]. However, a recent single-center observational study suggested that, among children 7–18 years old, 6MWD < 352 m and desaturation during the test (>5% for children with no shunt, >19% for children with a shunt) were associated with worse transplant-free survival [80].

Cardiopulmonary exercise testing in children over 7 years of age is useful to determine peak oxygen consumption, ventilator efficiency slope (VE/VCO2), and anaerobic threshold [81,82]. Ventilatory efficiency slope is significantly higher in patients with PAH, with an estimated increase of 7.2 for each increase in WHO class, and correlates strongly with invasive measures of disease severity including PAP, PVRI and outcome [83].

**Figure 9:**
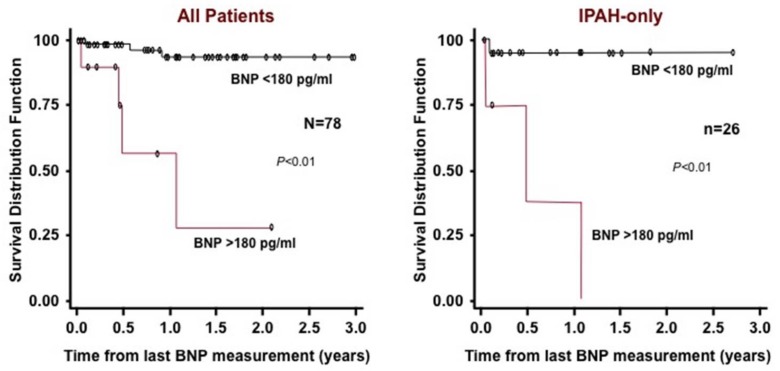
Kaplan–Meier survival curves for children with IPAH and PAH associated with CHD. Survival curves are shown for all patients (**left**) and for the subgroup of IPAH patients (**right**) categorized with either brain natriuretic peptide (BNP) > 180 pg/mL or < 180 pg/mL [85].

In adults, brain natriuretic peptide (BNP) is a useful tool to assess mortality risk, progression of the disease and response to therapy [84]. Recent studies in children have begun to identify usefulness of BNP and N-terminal–pro-brain natriuretic peptide (NT-proBNP) in pediatrics as well [85,86,87]. Change in BNP measurements over time typically trend with changes in classic hemodynamic and echocardiographic parameters of disease severity for children with PAH. In one study, patients with a BNP value > 180 pg/mL had worse survival compared to those with a BNP value < 180 (Figure 9). An NT-proBNP > 1200 ng/L portends a poor prognosis [65].

Recent large studies have attempted to validate potential biomarkers of prognosis and disease severity from multivariable models involving multiple modalities of testing currently used in standard clinical practice. For such biomarkers to be of clinical use, they must be associated with improved survival or another clinical endpoint. In the Netherlands national registry and a related meta-analysis, WHO FC (from clinical history), NT-proBNP (from a peripheral blood sample), mean right atrial pressure, cardiac index, pulmonary vascular resistance, pulmonary vasoreactivity (from cardiac catheterization), and TAPSE (from resting echocardiogram) were identified as variables for which improvement in response to therapy correlated with better survival [65,88].

## 7. Emerging Evaluation Techniques

Newer techniques, such as cardiac MRI and 3-dimensional echocardiography, offer the promise of evaluating right ventricle and pulmonary artery function in novel and more thorough ways. Total right ventricular afterload can be measured as pulmonary vascular input impedance and consists of both a pulsatile component (relating to vascular compliance versus stiffness) and a static component (PVR) [89,90,91]. In children, the pulsatile components of right ventricular afterload, represented by pulmonary arterial capacitance and pulmonary stroke volume index, can add important prognostic information to conventional static hemodynamic parameters [92,93,94]. RV stroke work (RVSW), the product of mean pulmonary artery pressure and stroke volume, is additionally of interest as it integrates information pertaining to contractility, afterload, and ventricular–vascular coupling. RVSW can be estimated in children with PAH by echocardiography or catheterization, and is significantly associated with abnormal WHO functional class, the need for atrial septostomy, as well as mortality [95,96]. Evaluation of MRI parameters in children with PAH has shown that right ventricular ejection fraction and left ventricular stroke volume index were most strongly predictive of survival on univariate analysis (2.6- and 2.5-fold increase in mortality for every 1-SD decrease, respectively) [97].

Simple, non-invasive tools to assess functional status may additionally offer prognostic value in PAH. Decreased physical activity, as measured by an actigraphy device worn on the wrist of adult PAH patients in the outpatient setting, has been shown to correlate with increased disease severity and worse prognosis [98]. A more recent study validated this finding in children with PAH, showing a positive correlation between actigraphy-measured physical activity, 6MWD, and improved functional class [99].

Studies of immune system function suggest that many aspects of inflammation are an important contributors to PAH in both children and adults [100,101]. Serum amyloid A-4 (an acute phase protein released in response to inflammatory stimuli) was 4-fold higher in children with poor outcome (death, initiation of intravenous prostacyclin) compared to those with good outcome (survival, discontinuation of intravenous prostacyclin) [102]. Blood level of Interleukin-6, a proinflammatory cytokine, is associated with the occurrence of an adverse event in pediatric PH [103]. Circulating fibrocytes and myeloid-derived suppressor cells (MDSCs), both integral to certain types of immune response, are increased in children with PAH compared to non-PAH controls [102]. High levels of tissue inhibitors of metalloproteinases-1 (TIMP-1, overexpressed by proinflammatory cells), and low levels of apolipoprotein-A1 (reduce levels of oxidized lipids and improve vascular disease), are strongly associated with outcome in pediatric PAH [104,105]. As of yet, none of these putative inflammatory biomarkers of disease severity are in routine clinical use and none have proven to date to be useful therapeutic targets.

## 8. Cardiac Catheterization and Acute Vasoreactivity Testing 

Cardiac catheterization as soon as is clinically safe after clinical suspicion of PH arises is essential to confirm the diagnosis, quantify any shunt lesions, calculate PVR, and perform acute vasoreactivity testing. Cardiac catheterization carries a greater risk in those children with baseline suprasystemic pulmonary arterial pressure compared to those with less severe PAH (Odds Ratio = 8.1, *p* = 0.02) [106,107]. In the Tracking Outcomes and Practice in Pediatric Pulmonary Hypertension (TOPP) registry of PAH patients, complications associated with heart catheterization were analyzed in a total of 908 studies; 554 were at diagnosis and 354 in follow-up. Complications were reported in 5.9% with five deaths (0.6%) considered related to catheterization, suggesting a higher rate of catheterization complications compared to adult studies of patients with PAH [54,108].

Acute pulmonary vasoreactivity testing at the time of cardiac catheterization is an important step in the diagnosis and risk stratification of PH. A recent consensus statement from the Pulmonary Vascular Research Institute (PVRI) has helped standardize the practice across centers [109]. To perform vasoreactivity testing, a short-acting vasodilator is given, most commonly as a combination of 20–40 parts per million of inhaled nitric oxide and oxygen and hemodynamics are re-assessed [110,111,112]. Use of inhaled prostacyclin, inhaled trepostinil, inhaled milrinone, inhaled nitroglycerin, intravenous adenosine, and intravenous prostacyclin have been reported as well.

**Figure 10:**
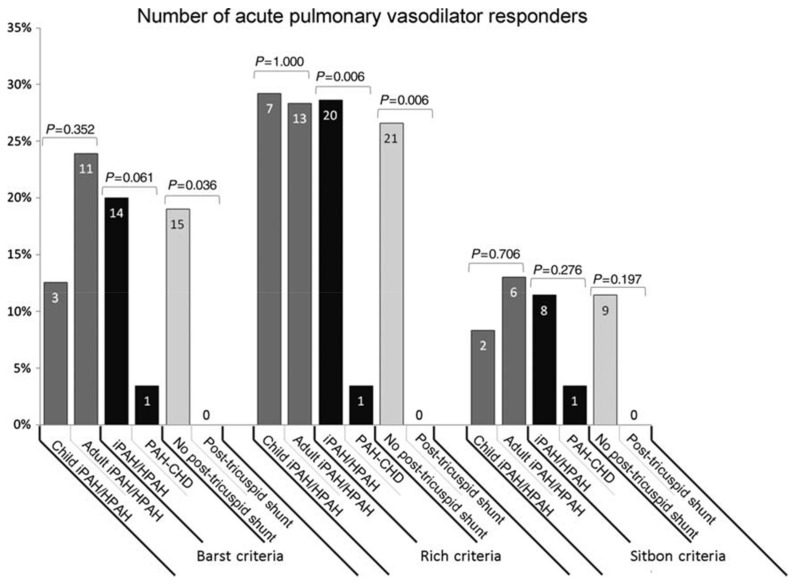
The number of acute pulmonary vasodilator responders according to the three criteria in use, in children vs. adults with idiopathic pulmonary arterial hypertension (IPAH)/hereditary pulmonary arterial hypertension (HPAH), IPAH/HPAH vs. pulmonary arterial hypertension associated with congenital heart disease, and patients without vs. with post-tricuspid shunt, respectively. Data presented as percentage of patient group (%) and patient numbers (indicated in bars). Comparison between groups performed using Fisher’s exact test. Note the few % of patients with PAH-CHD responding to acute vasodilator challenge [114].

Barst (1999) and Sitbon (2005) have each suggested potential strategies to classify patients as responders (positive test) or non-responders (negative test), but consensus remains elusive [110,113]. In the Barst criteria, vasoreactivity testing is positive if the patient demonstrates a >20% decrease in mean pulmonary artery (PA) pressure with improved PVR:SVR (systemic vascular resistance) ratio and unchanged or improved cardiac index. Whereas, in the Sitbon criteria, a patient is labeled as vasoreactive if they have a decrease in mean PA pressure of at least 10 mmHg and a nadir mean PA pressure under 40 mmHg with an unchanged or increased cardiac output. In a third proposed scheme, the PVRI suggests that patients must achieve PVR < 4.5 Woods units × M^2^ and “near normalization” of pulmonary artery pressures to be labeled as acute responders [109]. There was no difference in the number of responders in children with IPAH comparing the Barst or Sitbon criteria in a Netherlands study (Figure 10) [114]. Depending on criteria used and patient population included, the percentage of patients with a new diagnosis of IPAH who are acute responders is between 6% and 20% [3,18,114,115]. A recent registry study by Douwes et al. suggested improved survival in those children meeting the Sitbon criteria as acute responders [116].

## 9. Targeted Pharmacological Therapy for PAH

**Figure 11:**
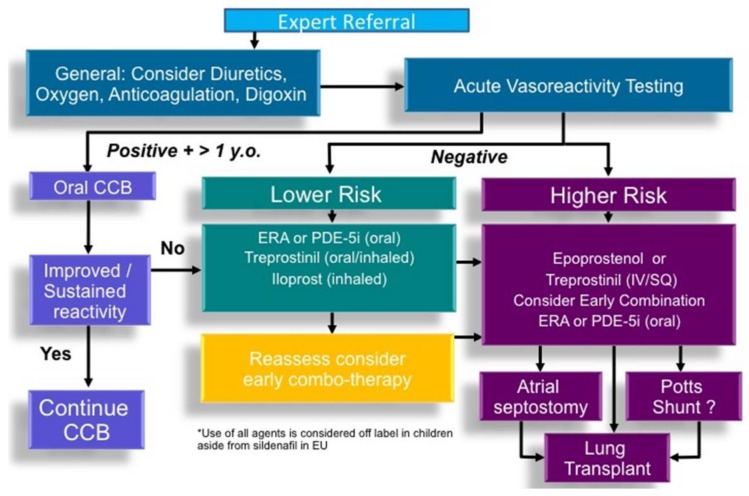
Adapted treatment algorithm proposed in the management of pediatric patients with idiopathic or heritable pulmonary arterial hypertension. This may be translatable to other patients with pulmonary hypertension. CCB, calcium channel blocker; ERA, endothelin receptor antagonist; PDE-5i, phosphodiesterase 5 inhibitor [3].

**Figure 12:**
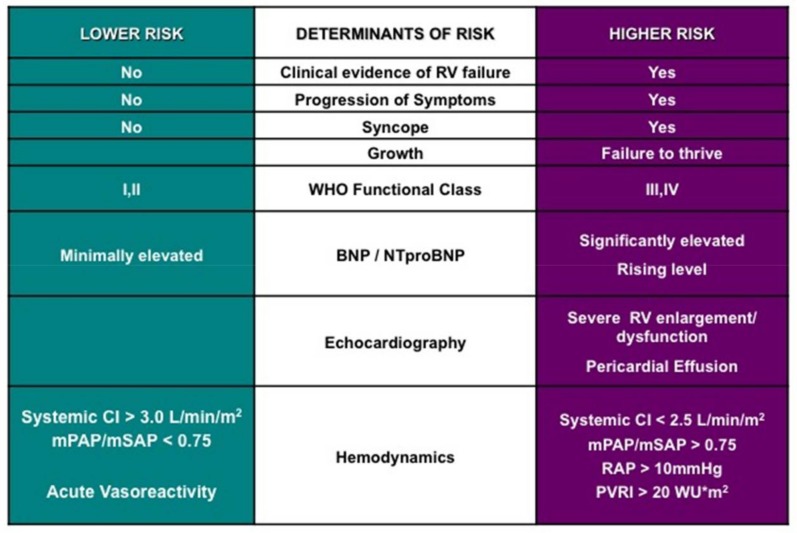
Risk factors that should be considered when planning therapeutic management options in pulmonary hypertension. CI—cardiac index; mPAp—mean pulmonary artery pressure; mSAp—mean systemic aortic pressure; NT-proBNP—N-terminal–pro-brain natriuretic peptide; PVRI—indexed pulmonary vascular resistance; RAP—right atrial pressure; RV—right ventricle; SBNP—serum brain natriuretic peptide [3].

Based on known mechanisms of action, three pathways of drugs have been extensively studied for the treatment of primarily IPAH in adults: prostanoids, which stimulate cAMP (epoprostenol, treprostinil, iloprost, beraprost), endothelin receptor antagonists, which block endothelin signaling (bosentan, ambrisentan, macitentan), and drugs which stimulate the nitric oxide-cyclic GMP system (phosphodiesterase inhibitors: sildenafil, tadalafil; soluble guanylate cyclase stimulators: riociguat). A pediatric-specific treatment algorithm, which applies mostly to children with IPAH, was developed at the World Symposium of Pulmonary Hypertension in Nice (2013) and a recent adaptation is presented incorporating newer pharmaceutical therapies and surgical approaches (Figure 11) [3]. When initiating therapy, certain patient-specific clinical factors should be considered to guide risk stratification (Figure 12).

### 9.1. Calcium Channel Blockers

Calcium channel antagonists are not used to evaluate acute vasoreactivity in the catheterization laboratory due to the risk of an acute decrease in cardiac output or a marked drop in systemic blood pressure. Similarly, elevated right atrial pressure and low cardiac output are contraindications to acute or chronic calcium channel blockade in PAH. Patients who do not have an acute vasodilatory response to short-acting agents and who are then placed on calcium channel blocker therapy are unlikely to benefit from this form of therapy [110]. Sixty to eighty percent of children with severe pulmonary hypertension are non-responsive to acute vasodilator testing, and therefore require therapy other than calcium channel antagonists (typically initiation of PAH-targeted therapy, described below). In those patients who are acutely responsive to either nitric oxide or prostacyclin, our preference is to perform a trial of calcium channel blocker mono-therapy. As previously noted, pediatric PAH patients who demonstrate acute vasoreactivity have favorable clinical outcomes compared to those who are not reactive [116]. However, children and adults treated with calcium channel blockers may lose this response over time and must be monitored carefully for sustained efficacy [110,117]. In cases where previously documented vasoreactivity is lost and PAH persists, we favor a strategy of transitioning patients from calcium channel blocker to PAH-targeted therapy.

### 9.2. Prostacyclins

Adults with IPAH and children with congenital heart disease demonstrate an imbalance in the biosynthesis of thromboxane A_2_ and prostacyclin [118,119]. Likewise, adults and children with severe pulmonary hypertension show diminished prostacyclin synthase expression in the lung vasculature [120]. Prostacyclin administered over the long term, utilizing intravenous epoprostenol, has shown to improve survival and quality of life in adults and children with idiopathic PAH [4,110,117,121,122,123].

Prostacyclin and prostacyclin analogues impact the cyclic-AMP pathway to reduce pulmonary artery pressure. Food and Drug Administration (FDA) approved in 1995, intravenous epoprostenol-prostacyclin was first used in the 1980s and continues to be the gold standard for the treatment of severe disease. Seventy-seven children diagnosed between 1982 and 1995 with idiopathic pulmonary arterial hypertension were followed up through 2002. Survival for all children treated with epoprostenol (*n* = 35) at 1, 5 and 10 years was 94%, 81% and 61%, respectively [117].

The prostacyclin analogue treprostinil was approved by the FDA initially for subcutaneous use (2002), followed by intravenous administration (2004), inhaled administration (2009), and oral treatment (2013). While subcutaneous treprostinil allows patients to remain free of central venous catheters, it can cause pain at the infusion site. Intravenous treprostinil requires central line access and continuous infusion, but may be used at room temperature, and has a half-life of four hours. Good long-term efficacy of subcutaneous treprostinil [124] and intravenous treprostinil [125] has been shown in adults with PAH. Intravenous treprostinil has fewer side effects overall than intravenous epoprostenol [126]. Prior studies suggested a higher rate of bacteremia in children and adults treated with intravenous treprostinil, but this may be decreased by protecting catheter connections, avoiding water on any connection, and use of a more basic buffer [127]. There are no studies comparing efficacy of the two medications. Subcutaneous treprostinil is well tolerated in many children with mild side effects [128,129]. Treprostinil has also been studied in an inhaled form and is reasonably well tolerated by affected children [130,131,132].

Oral treprostinil was shown to be effective as initial monotherapy treatment in adult PAH [133], but not as add-on therapy [134]. One report noted improved 6MWT distance and decreased pulmonary vascular resistance after initiating therapy in 37 treated adults (25 IPAH, 5 heritable PAH, 6 with connective tissue disease, 1 with CHD-PAH) [135]. In that study, 34 of 37 patients were able to target goal dosing with tolerable or no side effects. A recent publication have described early experiences with the oral prostacyclin analog selexipag in pediatric patients, targeting a goal dose of 1600 mcg twice per day [135,136]. That study noted similar results with 9 of 10 patients reaching goal dose successfully and a reported trend towards improved hemodynamics [136]. However, the long-term outcome in patients with severe disease remains to be determined. Future studies are needed to rigorously evaluate the efficacy of oral prostacyclin analog therapy in pediatrics, as oral prostacyclin analogs have not been shown to be as effective as continuous (intravenous or subcutaneous) prostacyclin in adult patients with severe disease.

Iloprost, an inhaled prostacyclin analogue, received approval for the treatment of PAH in the United States in December 2004. This medication is administered by nebulization 6–9 times a day. Iloprost requires patient cooperation with the treatment administration lasting 10–15 min [137], which is difficult for young children [138]. In the acute setting, inhaled iloprost lowers mean pulmonary artery pressure and improves systemic oxygen saturation [139]. Some children may develop reactive airways obstruction limiting usefulness of this therapy.

### 9.3. Endothelin Receptor Antagonists

The vasoconstrictor peptide endothelin (ET) is a target for treatment of pulmonary hypertension [140]. The endothelins are a family of isopeptides consisting of ET-1, ET-2 and ET-3. ET-1 is a potent vasoactive peptide produced primarily by the vascular endothelial cell, and secondarily by vascular smooth muscle cells [141]. Two receptor subtypes, ETA and ETB, mediate the activity of ET-1. ETA receptors on vascular smooth muscle primarily mediate vasoconstriction. ETB receptors on endothelial cells can both effect vasodilation via release of nitric oxide (NO) and prostacyclin (PGI2) and act as clearance receptors for circulating ET-1. ET-1 expression is increased in the pulmonary arteries of patients with pulmonary hypertension [142].

Bosentan, a dual ET receptor antagonist, lowers pulmonary artery pressure and resistance and improves exercise tolerance in adults with pulmonary arterial hypertension [140]. The first approved medication in its class, bosentan has been available since 2001 for the treatment of WHO functional Class III and IV patients over 12 years of age, and has recently shown beneficial effects in Class II patients [143]. A series of pediatric-specific randomized, placebo-controlled trials demonstrating efficacy and safety led the FDA to approve bosentan for pediatric use in September 2017 [144,145,146,147,148,149,150,151,152]. One recent pharmacology study comparing twice-daily versus three-times-daily dosing found that both regimens achieve acceptable drug levels, favoring 2 mg/kg/dose twice per day as the therapeutic target [153]. A prospective, noninterventional, internet-based post-marketing surveillance study evaluated safety and tolerability among younger (aged 2–11 years) and older (>12 years) bosentan-treated patients. Elevated aminotransferases were reported in 2.7% of children less than 12 years of age versus 7.8% in older patients. The discontinuation rate was 14.4% in young children versus 28.1% in patients over 12 years [147]. Prior studies have suggested a 12% rate of elevated aminotransferase levels in treated adult patients [151].

Macitentan, a dual endothelin-receptor antagonist with longer duration of action facilitating once-daily dosing, was FDA approved in 2013 for adults with PAH. In adult patients, macitentan reduced the time from the initiation of treatment to the first occurrence of a composite end point of death, atrial septostomy, lung transplantation, initiation of treatment with intravenous or subcutaneous prostanoids, or worsening of pulmonary arterial hypertension [154]. Pediatric-specific studies of macitentan are ongoing.

Selective ETA receptor blockade may benefit patients with pulmonary arterial hypertension by blocking the vasoconstrictor effects of ETA receptors while maintaining the vasodilator/clearance functions of ETB receptors. The FDA approved the ETA-specific antagonist ambrisentan for adult patients in June 2007. Adults showed significant improvements in 6-min walk distance and significant delay in clinical worsening on ambrisentan. The incidence of elevated hepatic aminotransferase levels was low at 2.8% [155]. Initial experience with ambrisentan in children suggests that treatment is safe, with similar pharmacokinetics and adverse reactions to those seen in adults, and effective at improving PAH in many patients [131,156].

### 9.4. Phosphodiesterase-5 Inhibitors and Soluble Guanylate Cyclase Stimulators

Phosphodiesterase-5 (PDE-5) is a membrane-bound protein that is localized to vascular smooth muscle with increased activity in models of PAH [157]. Specific PDE-5 inhibitors, such as sildenafil [158,159] and tadalafil [160,161,162], promote an increase in cGMP levels and thus effect pulmonary artery vasodilation and remodeling. Based on significant efficacy data and a well-tolerated side-effect profile, PDE-5 inhibitors are frequently used both as first-line outpatient therapy for PAH and acutely in critically ill patients. Although intravenous sildenafil may worsen oxygenation in the critical care setting due to increased ventilation/perfusion (V/Q) mismatching [163,164], it has been shown to prevent rebound PAH on withdrawal from inhaled NO [165,166].

**Figure 13:**
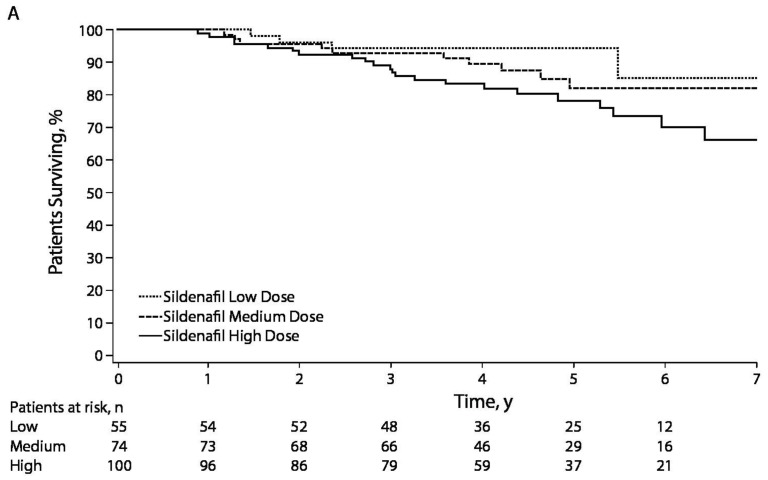
Kaplan–Meier estimated survival from start of sildenafil treatment in Sildenafil in Treatment-Naive Children, Aged 1 to 17 Years, With Pulmonary Arterial Hypertension (STARTS-1) and STARTS-2. Patients were censored at the last date they were known to be alive; if a patient received a transplant, he or she was censored the day before transplant. Patients at risk are those who are ongoing in the study or known to be alive at the specified time (i.e., not dead, not lost to follow-up, or not in study long enough to reach time point) [172].

Sildenafil has been approved for the treatment of WHO functional class II-IV PAH adult patients [158], and has been extensively studied in children with PAH [159,167,168,169,170]. In the 16-week, randomized, double-blind, placebo-controlled STARTS-1 study, the effects of oral sildenafil in pediatric PAH were evaluated [171]. Children (*n* = 235) with PAH (aged 1–17 years; ≥8 kg) received low-, medium- or high-dose sildenafil or placebo orally three times per day. The trial did not meet its primary endpoint (percentage change in pVO_2_ for the low, medium and high doses combined versus placebo was 7.7% ± 4.0%, 95% CI: –0.2% to 15.6%; *p* = 0.056) [171]. After the initial 16-week study, an extension trial (STARTS-2) was performed: patients in the low-, medium- and high-dose groups remained on their dose while patients in the placebo group were randomized to low, medium, or high dose [172]. By 3 years, the hazard ratio for mortality was 3.95 (95% confidence interval, 1.46–10.65) for high vs. low dose. Most patients who died had idiopathic or heritable PAH (76% vs. 33% overall) and baseline functional class III/IV disease (38% vs. 15% overall). Kaplan–Meier estimated that 3-year survival rates from the start of sildenafil were 94%, 93% and 88% for patients randomized to low-, medium- and high-dose sildenafil (Figure 13). Based on this, the data monitoring committee recommended that all patients down-titrate from the high dose. Review of the STARTS-1 and -2 by the FDA and the European Medicines Agency (EMA) resulted in disparate recommendations. Sildenafil was approved for pediatrics by the EMA in 2011, with a later warning to avoid use of the high dose. In August 2012, the FDA released a strong warning against the (chronic) use of sildenafil for pediatric patients (ages 1 through 17) with PAH (http://www.fda.gov/Safety/MedWatch/SafetyInformation/SafetyAlertsforHumanMedicalProducts/ucm317743.htm). In 2014, however, the FDA clarified the sildenafil warning, stating that there may be situations in which the risk–benefit profile may be acceptable in individual children, and that sildenafil is still not recommended in children with PAH. (http://www.fda.gov/drugs/drugsafety/ucm317123.htm).

Tadalalfil, another selective PDE-5 inhibitor, has a longer duration of action allowing for once-daily dosing. Data for tadalafil in pediatrics are limited. In one study of 29 children with PAH switched from sildenafil to tadalafil for convenience of dosing, the change was well tolerated (2 children discontinued due to headaches or allergic reaction) [162]. The average dose of sildenafil was 3.4 +/− 1.1 mg/kg/day, and that of tadalafil was 1.0 +/− 0.4 mg/kg/day. For 14 of the 29 patients undergoing repeat catheterization, statistically significant improvements in PA pressure and PVR were observed after transition from sildenafil to tadalafil. A study of tadalafil is underway in children.

**Figure 14:**
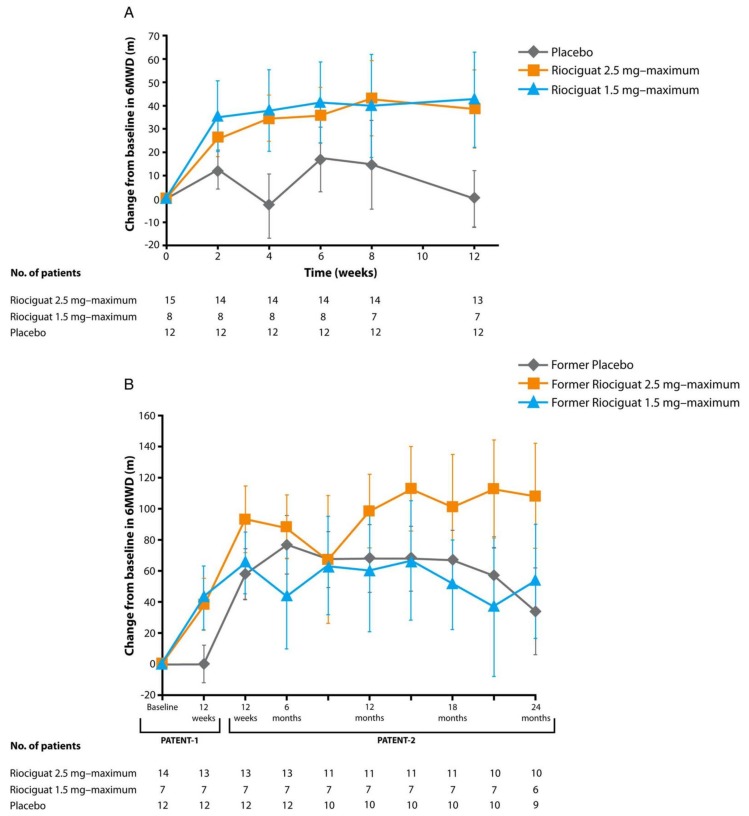
Change from baseline in 6-min walking distance (6MWD) in the subgroup of patients with pulmonary arterial hypertension associated with congenital heart disease in Pulmonary Arterial hyperTENsion sGC-stimulator Trial-1 (PATENT-1; (**A**) and PATENT-2 (**B**)). Data are observed values (mean ± SEM) [175].

Riociguat, a direct oral soluble guanylate cyclase (sGC) stimulator, increases cGMP directly in a non-NO dependent manner and also increases the sensitivity of sGC to NO [173]. Riociguat was approved by the FDA in 2013 for treatment of adult PAH [174] and is the first FDA-approved drug for the treatment of chronic thromboembolic PH [174]. In the PATENT-1 and -2 trials, riociguat was well tolerated in patients with repaired PAH-CHD and treated subjects demonstrated improved 6MWD, PVR, WHO FC and NT-proBNP (Figure 14) [175]. A recent single-center case report also described a patient with severe IPAH who experienced significant improvement in PVR and WHO FC after changing from sildenafil to riociguat [176]. A phase 3 safety and tolerability trial of riociguat, the PATENT-CHILD study, is currently enrolling children with PAH. (Clinicaltrials.gov identifier NCT02562235).

### 9.5. Combination Therapy

**Figure 15:**
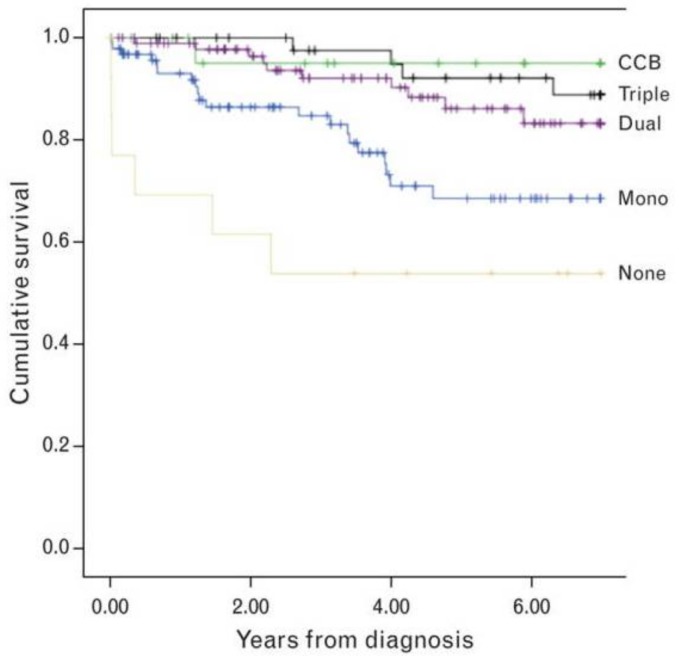
Survival according to extent of pulmonary hypertension therapy in 275 recently diagnosed consecutive pediatric PAH patients at 3 referral centers between 2000 and 2010. Survival improves on combination therapy for pulmonary hypertension over monotherapy. CCB, calcium channel blocker [4].

Targeting multiple pathways simultaneously, combination therapy is the standard treatment practice employed for many patients with more severe disease. Between 2000 and 2010, pediatric patients with PAH were compared among 3 centers. Treatment with PAH-targeted combination therapy during the study period was independently and strongly associated with improved survival compared to monotherapy (Figure 15) [4]. In the AMBITION trial, the risk of the primary end point of the first event of clinical failure was 50% lower among participants who received initial combination therapy with ambrisentan and tadalafil than among those who received monotherapy with either drug [177]. A more recent, retrospective study of 97 patients started on dual therapy (PDE-5 inhibitor + ET receptor antagonist) at diagnosis demonstrated good tolerability and improved survival when compared to expected longevity based on historical registry data [178].

## 10. Atrial Septostomy and Potts Shunt for Refractory PAH

**Figure 16:**
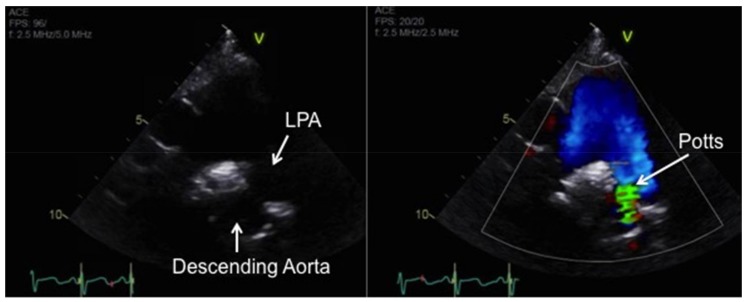
Echocardiogram of the Potts shunt in a patient with severe IPAH by 2D imaging (**left**) and with color Doppler (**right**). LPA, left pulmonary artery.

The general indications for atrial septostomy include severe pulmonary hypertension, syncope, intractable heart failure refractory to chronic PAH-targeted therapy, and symptomatic low cardiac output state [179,180]. Risks associated with this procedure include worsening of hypoxemia with resultant right ventricular ischemia, worsening right ventricular failure, increased left atrial pressure, and pulmonary edema. We favor a graded balloon dilation approach utilizing intra-procedure echo guidance and saturation monitoring to determine the adequacy of shunting. A Potts shunt—connection of the left pulmonary artery to descending aorta (Figure 16)—can be considered in cases of severe, refractory PAH with suprasystemic PA pressures and adequate RV function to allow an immediate reduction in right ventricular afterload [10,181,182,183]. The choice of initial atrial septostomy versus Potts shunt for patients failing medical therapy is a source of ongoing debate.

## 11. Transplantation

For patients who do not respond to prolonged treatment with PAH-targeted therapy, lung transplantation should be considered [184,185,186]. IPAH is the second most-common indication for lung transplant in pediatric patients overall, and is the most common indication among children aged 1–5 years [187,188]. Overall survival following pediatric lung transplant is similar to that encountered in adult patients, with recent registry data indicating a median survival of 4.9 years [188,189,190]. The most common causes of early post-transplant death include graft failure, technical issues and infection. Infection and bronchiolitis obliterans syndrome are the most common causes of late death after transplant.

## 12. Adjunctive Therapy

A number of adjunctive therapy options are employed to treat right ventricular failure and prevent the frequently encountered complications of PH. Digoxin has been tried in the presence of right ventricular failure, although without positive data in children. Many providers maintain patients on warfarin or other antithrombotic agents to prevent thrombosis in situ, although specific data and indications in the pediatric population are lacking. Clinical experience dictates that anticoagulation is most often used in children with a hypercoagulable state, those with severe IPAH, and those with a central venous line for intravenous prostanoid therapy. In adults and children with IPAH who receive anticoagulation, warfarin is typically dosed to target an INR of 1.5 to 2 [191]. Diuretics are used to treat peripheral edema or ascites in the presence of right heart failure, but excessive diuresis should be avoided.

Preventive medicine is of particular importance for patients with severe disease. Careful attention to respiratory tract infections with good supportive care is required as pneumonitis may worsen alveolar hypoxia. Routine influenza vaccination as well as pneumococcal vaccination is recommended. We recommend against the use of decongestants with pseudoephedrine or other stimulant-type medications as these have been associated with PAH [192]. In children who require the use of oral contraceptive agents either for prevention of pregnancy or for regulation of menses, we recommend agents that have no estrogen content.

Among non-pharmacologic options, intermittent home pulse oximetry monitoring and polysomnography are indicated for patients with severe PH; chronic hypoxemia and nighttime desaturation should be aggressively treated. While oxygen therapy is not used as a mainstay of therapy in children with normal daytime saturations, in the presence of resting hypoxemia chronic supplemental oxygen may be helpful.

## 13. Summary

Children with PH are a heterogeneous group with a wide range of ages at diagnosis, disease severities, and underlying etiologies. At diagnosis, a thorough evaluation for secondary causes of PAH and a cardiac catheterization with acute vasoreactivity testing should be completed. Although treatment of children with PAH is based primarily on extrapolation of adult data and clinical experience, therapeutic options and survival have dramatically improved over the past two decades. Despite a new treatment algorithm for children to guide providers (World Symposium of PAH, Nice, 2013), more pediatric-specific studies are needed to optimize therapy for affected children.
